# Capripoxvirus Infections in Ruminants: A Review

**DOI:** 10.3390/microorganisms9050902

**Published:** 2021-04-23

**Authors:** Jihane Hamdi, Henry Munyanduki, Khalid Omari Tadlaoui, Mehdi El Harrak, Ouafaa Fassi Fihri

**Affiliations:** 1 Department of Research and Development, Multi-Chemical Industry Santé Animale, Lot. 157, Z I, Sud-Ouest (ERAC) B.P., 278, Mohammedia 28810, Morocco; k.tadlaoui@mci-santeanimale.com (K.O.T.); m.elharrak@mci-santeanimale.com (M.E.H.); 2 Pirbright Institute, Ash Rd, Pirbright, Woking GU24 0NF, UK; Henry.Munyanduki@pirbright.ac.uk; 3 Department of Microbiology, Immunology and Contagious Diseases, Agronomic and Veterinary Institute Hassan II, Madinat Al Irfane, Rabat 6202, Morocco; o.fassifihri@iav.ac.ma

**Keywords:** capripoxvirus, live, inactivated, combined, vaccines, cross protection

## Abstract

Lumpy skin disease, sheeppox, and goatpox are notifiable diseases of cattle, sheep, and goats, respectively, caused by viruses of the Capripoxvirus genus. They are responsible for both direct and indirect financial losses. These losses arise through animal mortality, morbidity cost of vaccinations, and constraints to animals and animal products’ trade. Control and eradication of capripoxviruses depend on early detection of outbreaks, vector control, strict animal movement, and vaccination which remains the most effective means of control. To date, live attenuated vaccines are widely used; however, conferred protection remains controversial. Many vaccines have been associated with adverse reactions and incomplete protection in sheep, goats, and cattle. Many combination- and recombinant-based vaccines have also been developed. Here, we review capripoxvirus infections and the immunity conferred against capripoxviruses by their respective vaccines for each ruminant species. We also review their related cross protection to heterologous infections.

## 1. Introduction

Sheeppox, goatpox, and lumpy skin disease (LSD) are notifiable diseases of cattle, sheep, and goats, respectively. Their causative agents are viruses belonging to the Capripoxvirus genus of the Poxviridae family [1]. They are economically important because they cause fever, emaciation, weight loss, drop in milk production, and skin damage [2].

**Figure 1:**
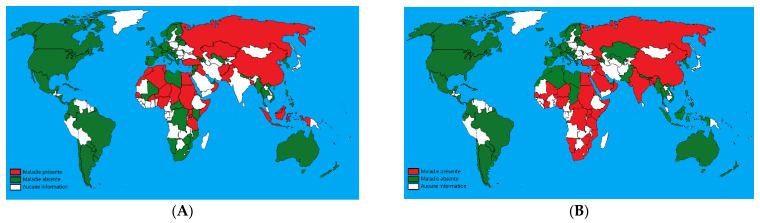
(**A**): Geographical distribution of sheeppox; (**B**): geographical distribution of lumpy skin disease (Adapted from World Animal Health Information Database (WAHIS), 2020). Green: absence of the disease; red: presence of the disease; white: unavailable data.

The three viruses are endemic in Africa, Middle East, central Asia, and the Indian subcontinent. LSD is also present in many parts of Europe, the Balkans (namely Bulgaria, Serbia, and Albania) [3], and in 2019, incursions of LSD into India, China, and Bangladesh were reported (Figure 1) [4].

Capripoxviruses have the characteristic of using direct or indirect means to infect their hosts. Transmission can occur either by direct contact, through aerosols released by infected hosts, or indirectly, through the environment and infected vectors [5]. However, the routes of transmission can vary from virus to virus, even within the same genus [6]. The sheeppox (SPV) and goatpox (GPV) viruses are mainly found in oral, nasal, or ocular secretions, and transmission between animals occurs by direct contact through inhalation of aerosols or by indirect transmission [7,8]). For the LSD virus, authors report mainly mechanical transmission by insects such as *Aedes aegypti* mosquitoes, *ixodid* ticks, *Stomoxys calcitrans*, and the tabanids *Haematopota* spp. [9,10,11,12,13,14].

Clinical signs of Capripoxvirus diseases are variable depending on the individual host’s susceptibility, the virus species, and virulence of the viral strain. These can be mild, severe, or sometimes fatal. Usually, signs include fever, enlarged lymph nodes, oculonasal discharge, and eruption in the skin and mucous membranes [15,16,17].

Control and eradication of diseases due to capripoxviruses depend on vector control, early detection of outbreaks, restrictions on animal movement, and vaccination [18]. Attenuated vaccines are mainly used and commercially available; however, the conferred protection remains controversial because these vaccines may be ineffective or cause mild reactions [19,20,21]). Inactivated vaccines are safe, stable, and allow combinations with other antigens to make polyvalent vaccines, and they can be applied in disease-free countries. Even though a few inactivated capripoxvirus vaccines exist, sheeppox and LSDV vaccine were shown to offer full protection against their respective virulent challenge strains [22,23,24]. Combined SPV/GPV vaccines with other antigens have been reported to protect small ruminants against two infections. Recombinant capripoxvirus-vectored vaccines are also reported in the literature.

Here, we review the capripoxvirus immunity and protection conferred by different available vaccines.

## 2. Importance

The three diseases of the Capripoxvirus genus are economically important and classified by the World Organisation for Animal Health (OIE) as notifiable diseases because of their rapid spread and the significant economic losses they can cause. The three viruses also present obstacles to genetic improvement in small ruminants and cattle breeding.

Sheeppox and goatpox cause annual losses associated with a decrease in milk production and weight gain, damage to wool and hides, and high mortalities in lambs and kids [3,25]). In a study conducted in India, Garner et al. (2000) estimated income losses up to 30–43% of the total annual revenue and reported that the recovery of a herd from an epidemic may take up to six years [26].

Vaccination campaigns, restrictions on animal movements, and restrictions on the trade of live animals or their products also contribute to significant financial losses.

The lumpy skin disease attack rate is around 10% in endemic areas; however, it can reach 85% in an affected herd if no preventive measures are applied [27]). The impact of the disease is significant during peak lactation due to fever and secondary mastitis in infected animals [3]. Deep lesions recorded in the skin leave permanent scars which reduce the quality of skins destined for the leather industry [28]. Cattle rearing for slaughter is also affected due to wasting and long convalescence of infected cattle [29]. The control procedures in the event of a disease outbreak, the quarantine of neighbouring farms, and the costs borne by the breeders in the control of the disease are additional costs that impact the economy [30,31]. Internationally, LSDV epidemics result in restrictions in the cattle trade and can cause considerable losses [32]. Restrictions apply to live animals, meat, leather, and dairy products [31].

In addition, capripox diseases are restrictive for genetic improvement of livestock because high-milk producing cattle are more severely affected by the infection, compared to local breeds [3,33].

## 3. Aetiology

Sheeppox, goatpox, and lumpy skin disease of cattle result from infection by SPV, GPV, and LSDV which are members of Capripoxvirus genus within *Poxviridae* family [6]. Even though they are serologically indistinguishable, they may be differentiated using molecular analysis [28,34,35].

Poxviruses are ovoid-shaped and characterised by a complex structure. They are 300 to 400 nm long [36] and can be identified using electron microscopy [37]. The virion takes the form of a central core containing the genome and the various viral proteins. The core and the two lateral bodies are surrounded by a capsid [38,39].

As they appeared thousands of years ago, poxviruses have seen their genomes evolve by the gain or loss of genes through duplication and horizontal transfer of genes [40,41,42]. Capripoxviruses are double-stranded DNA viruses whose ends are covalently linked to form a hairpin structure [43]. The central region of the genome contains conserved genes which encode viral replication and structural protein synthesis. Regions at the ends, called inverted terminal repeat sequences (ITR), are made up of non-essential genes involved in virulence [44].

Capripoxviruses genomes contain a high percentage of adenine and thymine [45] and share 147 putative genes which encode proteins of 53 to 2027 amino acids (AA). These proteins are involved in replication, structure, virulence, and host range functions. The molecular analysis of the three capripoxviruses has been studied by several authors. Tulman et al. (2002) sequenced and analysed the complete genomes of several strains of SPV, GPV, and LSDV and reported the presence of an ancestral virus, related to LSDV [46]. Biswas et al. (2019) analysed 36 different strains and found a loss of 5 Open Reading Frames (ORF) in the SPV/GPV lineage [35]. Additionally, Rouby et al. (2018) [47] reported deletion of 21 nucleotides in the RPO30 gene of SPV, while the analysis of a partial fragment of the B22R gene showed a deletion in the SPV Romania strain, compared to GPV and LSD (Chibssa et al., 2019) [48]. These findings have confirmed that GPV is more closely related to LSDV than SPV [49,50].

## 4. Clinical Signs

The incubation period is 4 to 8 days for SPV and 4 to 15 days for GPV and LSD. After its entry into the host, the virus replicates in the tissues. It is localised in the lymph nodes three to four days after a primary viraemia. The virus then spreads throughout the body and affects the liver, lungs, and spleen. Nodules appear 7 to 19 days post-infection. After the appearance of skin lesions in affected animals, they may develop conjunctivitis, rhinitis, and lymphadenopathy, in the prescapular lymph nodes. Excessive salivation can occur after an infection [17].

The clinical signs of SPV and GPV are similar and can be mild or severe, sometimes fatal. They are variable depending on the individual susceptibility of the host and the viral strain; varying degree of severity has been reported in goats of same age and breed, infected with the same strain [15]. The diseases are more serious in young animals than in adults which usually have a mild form. These signs usually include fever, which can reach 40 to 42 °C, enlarged lymph nodes, oculonasal discharge, and damage to the skin and mucous membranes. Skin lesions usually begin as erythematous macules that harden to form papules measuring between 0.5 to 1.5 cm in diameter which are usually devoid of fluid. The papules then form pustules and scabs following the necrosis of tissues [51]. These lesions are most often found in hairless areas of the animal’s body such as the armpits, face, ears, eyelids, and inguinal area, but they can spread all over the body in severe cases [52]. On the mucous membranes, especially on the mouth, nostrils, eyes, vagina, and foreskin, the lesions can ulcerate or necrose. These lesions cause a lack of appetite, ptyalism, and mucopurulent discharge. Lesions in the eyes and eyelids can cause conjunctivitis and blepharitis. Damage to the intestinal tract or respiratory system can lead to diarrhoea, emaciation, or coughing, and abortion and pneumonia can also occur.

In the benign form, the lesions are rather localised under the tail or at the level of the ears. Lesions are visible to the naked eye but may also require palpation, especially in animals with thick fleece. Skin lesions are slow to heal and can cause permanent scarring. They can also be accompanied by bacterial complications or myases [53]. A nodular form, called ‘stone pox’, has also been described [51]. It consists of the appearance of nodules all over the thickness of the skin, which eventually become necrotic. After necrosis, the nodules break off and leave ulcerative lesions or scabs that heal [51].

Lumpy skin disease is characterised by the appearance of localised or generalised nodules, with deterioration of the general condition depending on the severity of the disease. Natural resistance to LSDV has been reported in cattle. In experimental infections, only half or two-thirds of infected animals may show clinical symptoms of the disease, although all animals are viraemic [3,54,55]. Gari et al. (2015) and Hamdi et al. (2020) also described the development of clinical disease in only three out of five challenged control animals during their experiments [56,57].

The disease manifests as a biphasic febrile syndrome with a peak a few days after infection and a second at day 10 post-infection. During this phase, the animals generate salivary, nasal, and lacrimal secretions and eye disorders. Lymphadenopathy is particularly observed in the subscapular and precrural lymph nodes. A second febrile syndrome appears for 4 to 10 days after the first and is accompanied by skin nodules, pathognomonic signs of the disease [31]. The classic form is characterised by hyperthermia and localised or disseminated nodules on the skin, accompanied by respiratory disorders, adenomegaly anorexia, and dysgalactia. Lesions on the skin appear as macules that turn into papules and then into hard, rounded, painless nodules. These nodules vary in size between 0.5 and 5 cm in diameter. Their number varies from 1 to 100 nodules, and they are localised on the face, the neck, the limbs, the flanks, the udder and its teats, the scrotum, the perineum and on the oral, nasal, ocular, vulvar mucous membranes or foreskin [58]. The nodules resorb and scar, which leads to leather depreciation. This form is accompanied by conjunctivitis and keratitis which can progress to blindness [58]. Reproduction and milk production are affected and abortions are also common [59].

In the mild form, cattle exhibit small nodules that heal quickly with scarring in three to six weeks, without affecting the general condition. The nodules are perceptible to the touch or spotted in the hairless areas of the bovine (at the level of the vulva, the udder, the muzzle, and the perineum).

## 5. Immunity

The immune response to an infectious agent is characterised by the coexistence of innate and adaptive immunity and is particularly complex in poxvirus infections. Each component of the immune system plays a vital role in the segregation and elimination of the virus [60].

Innate immunity occurs early and comprises anatomic, physiologic, endocytic, and inflammatory barriers [61]. It relies on the interaction between pattern recognition receptors (PRRs) and pathogen-associated molecular patterns (PAMPs) and serves as the first line of defence to the pathogen. The PRRs are expressed in numerous cells including phagocytes (macrophages and neutrophils), dendritic cells, mast cells, basophils, eosinophils, innate lymphoid cells, and natural killer (NK) cells which produce interferon gamma [60]. Unlike the role of cellular immunity and of antibodies, the role of these cells has not been fully described in capripoxviruses [31].

The immune response is subsequently adaptive and includes B cells and T cells. In the orthopoxvirus group, humoral immunity has been reported to confer protection from reinfection against poxviruses, while poxvirus clearance is induced by both humoral and cellular responses [3,62]. B cells are responsible for humoral-mediated immunity—they recognise the antigen, produce specific antibodies, and can also act as Antigen-presenting cells (APC) [63,64]. While the humoral-mediated immunity is known to provide protection against capripoxviruses, it has been reported that only 34% to 65% of cattle produce antibodies following vaccination with live attenuated LSD vaccine [23,65,66]. It has also been reported that small ruminants could be protected against capripoxvirus even with a neutralisation index below 0.5 log_10_ and no precipitating antibodies [67]. However, although circulating antibodies may limit the spread of the virus, they do not prevent replication of the virus at the site of infection [68].

These findings indicate that cell-mediated immune response is of importance in poxvirus infection and is necessary at the early stage of infection [69].

Cell-mediated immunity is crucial against capripoxvirus infections and contributes significantly to the protection of the host [3,70,71]. The most important role is played by T cells, which are produced in the bone marrow and complete their maturation in the thymus. They require the action of APCs to recognise a specific antigen, unlike B cells [72,73]. Although T cells are differentiated into poxvirus-specific cytotoxic T (LT) cells (CD8+) and helper (LTh) cells (CD4+) (which are important for the maturation of B cells), their role in protection against capripoxviruses has not completely been elucidated [3]. Their contribution to immunity against other poxviruses such as ectromelia, monkeypox, and vaccinia viruses has been studied in mice, rhesus macaques, and humans [74,75,76,77]. Some authors also reported the development of generalised vaccinia in individuals with abnormalities of T-cell function, in an immunocompromised individual, whilst patients with congenital agammaglobulinemia did not develop the disease [78,79,80]. The induction of cell-mediated response is dependent on the immunological status of the individuals. The T-cell response occurs earlier in non-naïve than in naïve persons, due to the presence of memory T cells [81]. T-cell immunity remains stable for decades; some authors reported a decrease of T cells 8 to 15 years after vaccination against smallpox [82], while others reported the presence of T-cell memory of up to 30 years after vaccination [83]. B-cell memory to smallpox has been reported to persist for 50 to 90 years after vaccination [82,84,85].

The passive transfer of maternal capripoxvirus antibodies has been investigated by several authors. Agianniotaki et al. (2018) [86] detected antibodies in calves after feeding with colostrum until three months of age, and Milovanovic et al. (2019) [65] showed, through Indirect Fluorescent Antibody Test (IFAT), Virus Neutralization Test (VNT), and Enzyme-linked Immunoassay (ELISA), that cows provide colostral antibodies to their calves. Weiss (1968) also reported the persistence of antibodies in calves after colostrum feeding for up to six months [87]. In sheep, the presence of maternal antibodies has been detected in lambs from ewes vaccinated with SPPV vaccine [88,89], and in a challenge trial with the Romania strain, it has been reported that all lambs born displayed neutralising antibodies [90].

Capripoxvirus antigens involved in the humoral response are not known; however, studies on orthopoxviruses showed the presence of nine specific B-cell epitopes. Five of these, namely, H3, B5R, A27, D8, and L1R, induced protective neutralising responses and one-A33-induced protection with non-neutralising antibodies in mice against vaccinia virus [3,62,91,92,93].

## 6. Vaccination against Capripoxviruses

Control and eradication of capripoxvirus infections depend on veterinary services, farm holders’ sensitisation, and early detection of disease in a geographic area. The control also requires the slaughter of infected animals, monitoring of movements, and establishment of a quarantine system [3,94]. Vaccination remains the best way to control infection and transmission of capripoxviruses; however, there are factors that can affect its effectiveness. Vaccination during an epidemic, the reuse of needles, improper administration of the vaccine, and disruption of the cold chain can affect the protection conferred by the administered vaccine [31,95,96].

Live attenuated vaccines are mostly used to control capripoxvirus infections [31]. Several strains of SPV/GPV are cited in the literature. They are named on the basis of the place of isolation (Jaipur [97], Uttarkashi [98], Romania [88], Cairo [94], Chinese, etc). These strains have been attenuated by passages through different cell culture systems or on embryonated eggs [99,100] (Table 1).

Sheep are mostly vaccinated with Romania, Bakirkoy, Yugoslavian RM65, KSGP O 240, and KSGP O 180 strains, goats with Gorgan, Mysore, Uttarkashi, and KSGP O 240 strains, while cattle are vaccinated with Neethling, KSGP O 240 and KSGP O 180, Romania, Bakirkoy, and Gorgan strains.

In North Africa and the Middle East, the Romania strain is mostly used to protect sheep, while in East Africa, the KSGP O 240 and KSGP O 180 are commonly used. In Turkey, vaccination of sheep is conducted using the Bakirkoy strain, while Iran uses the RM65 strain [112,113,114,115].

The attenuated Gorgan strain is used in Iran and the Middle East to protect against goatpox infection, while in India, vaccination is carried out using Uttarkashi and Mysore strains of caprine origin [106,107,116].

The KSGP O 240 vaccine is mainly used against SPV and GPV in East Africa, although molecular characterisation has identified it as an LSD virus [49,117,118]. This vaccine, developed by Davies (1976) [111], has been tested in sheep and goats and have been shown to be protective against both SPV and GPV virulent strains [112]. Additionally, the two strains—KSGP O 240 and KSGP O 180 (Kenyan origin)—have been used for a long time against capripoxviruses, especially against SPV. However, molecular characterisation using the RPO30 and GPCR genes of both strains revealed that they were actually LSDV strains [118]. Both strains have been successfully used for years in small ruminants.

To protect cattle against LSD, the Neethling strain is the most commonly used worldwide in endemic countries. The strain has been attenuated by 61 serial passages of a field isolate and then through 20 passages on the chorioallantoic membrane (CAM) and three passages on renal primary cells. The strain was then passed 10 times on Madin Darby Bovine Kidney (MDBK) cells, then five times in primary bovine testis cells by Weiss (1968) [87,99,119]. In addition to the original Neethling strain, other derivative strains with similar sequences (99% homology) were used as commercial vaccines [120] The Neethling strain has been shown to be effective in cattle; however, it can cause adverse inflammation at the injection site, accompanied by fever and decreased milk production [121]. The strain can also cause symptoms similar to the disease with lower intensity, called ‘Neethling disease’. Several studies have reported the development of adverse reactions after the vaccination of cattle against LSDV. According to Ben-Gera et al. (2015) [21], the LSD Neethling vaccine administered to cattle induced adverse effects with a very low incidence (0.38%). In Croatia, a free-disease country, the vaccine exhibited adverse effects in 0.09% of vaccinated cattle [122]). As these effects have been reported by breeders, the data can be underreported [123]. Agianniotaki et al. (2017) [124] were able to identify the vaccine virus in cattle vaccinated with Neethling strain that showed mild clinical signs of LSDV. Collected nodules, mainly from the head and neck, were smaller and more superficial than those found in animals infected with virulent LSDV and were not accompanied by fever, discharge, and enlarged lymph nodes that characterise the disease. Similarly, in a study in Greece carried out by Katsoulos et al. (2018) [121], skin lesions in the form of small nodules (<0.5 cm) were observed in 19 among 215 vaccinated animals. Bedekovic et al. (2017) [125] were able to detect the vaccine virus in the nodules, blood, and milk of cattle vaccinated with Neethling vaccines.

Two strains—KSGP O 240 and KSGP O 80—have also been used to protect cattle against LSD in the horn of Africa and Israel. Poor protection was induced when used in cattle and widespread pathognomonic reactions to LSD were reported in dairy cattle vaccinated with KSGP strain O–240 [20,126]. Salib and Osman (2011) [127] reported that an LSD epidemic occurred in Egypt although cattle were vaccinated with the KSGP O-240 vaccine. Similarly, in Ethiopia, a morbidity rate of 22.9% was reported in cattle vaccinated with the same strain [20]. However, the vaccine failure could be linked to insufficient vaccination coverage and the quality of vaccines [31]. The onset of the disease in cattle following vaccination with the KSGP strain could be explained by the insufficient attenuation of strains used. The KSGP O-180 strain was attenuated by 18 passages on muscle cells, while the KSGP O-240 strain was attenuated by only six passages [3,110,128].

In general, homologous vaccines offer good protection and are able to control the diseases when vaccination coverage reaches 75% [123]. Depending on the strain and the animal sensitivity, neutralising antibodies appear 14 days post-infection or vaccination and peaks between 28- and 35-days post-vaccination. Vaccinated animals can be protected even when antibodies are no longer detectable because of the dominance of cell-mediated immunity.

There is little information regarding the duration of immunity to capripox vaccines. Some authors report protection for at least two years after immunisation with live vaccines against SPV [105,116,129]; others have been able to demonstrate protection against challenge with goatpox virus more than four years after vaccination [130].

Few inactivated vaccines have been developed to protect areas at risk or in case of incursion in a free-disease country. The protection conferred with inactivated capripoxviruses has been described by Awad et al. (2003) as ‘non-solid and of short duration’, requiring a booster every six months [131]. More recent studies, however, showed complete protection after the challenge and persistence of antibodies for a longer time period. In a sheep study, a group of 16 sheep was immunised with an inactivated and live attenuated vaccine. The animals were monitored for serological responses and challenged with a virulent strain. All vaccinated sheep were protected against experimental infection and developed antibodies that lasted up to nine months post-vaccination [22]. In a cattle experiment, cattle were immunised using the inactivated Neethling vaccine. A high percentage of reactors had antibodies elicited and protected cattle against challenge. The vaccine was also tested in the field and showed at least 80% seroconversion [23]. A second LSD inactivated vaccine was tested recently in cattle and showed complete protection against challenge [24].

The low vaccination coverage is linked to poor infrastructure with limited access to flocks. It has been reported that the use of a bivalent vaccine that protects against two infections in one injection would minimise the cost of vaccination, overcome the constraints of multiple injections, and allow a large vaccination coverage. Among the combined vaccines that have been developed, a formalin-inactivated SPV vaccine with vaccination against anthrax and clostridium infections has been tested [132]. In another assay, the SPV vaccine was combined with anthrax [133]. Recently, many authors developed and tested the combination of SPV with Peste des Petits Ruminants (PPR) against both diseases. The SPV–PPR combined vaccines have been subsequently used at a large scale in Morocco and other African countries [113,134,135,136].

The use of poxviruses as vaccine vectors in recombinant vaccines presents many advantages such as the large size of the viral genome (140–300 kbp) which can contain up to 25,000 bases of foreign DNA, their thermal stability, and their replication in the cytoplasm of infected cells without integration into the host genome. Several recombinant capripoxvirus-vectored vaccines have been generated using genes of Rift Valley fever [137,138], peste des petits ruminants [139,140,141,142], rinderpest [143], bluetongue [144,145]), foot-and-mouth disease virus Mp1-2A polyprotein [146], EG95 antigens from Echinococcus granulosus [147], and OMP25 outer membrane protein from Brucella [148]. Developed recombinant capripox vaccines have not yet been used at a large scale in the field despite having the ability to differentiate them between infected and vaccinated animals (DIVA).

Different techniques are used to evaluate post-vaccination response. The humoral response is investigated by VNT and ELISA, which could be used for mass screening [65,149,150]). However, only a challenge can determine the protection against infection with a capripox [67]. The immunoperoxidase assay has also been developed for the detection of antibodies against LSDV, SPPV, and GTPV [151]. Other techniques such as Western Blot, counter–immunoelectrophoresis test (CIE), or agar gel precipitation test have also been described for the detection of anti-capripox antibodies [152]. The evaluation of the cell-mediated response is carried out by the lymphocyte proliferation is carried through MTT (3-(4,5-dimethylthiazol-2-yl)-2,5-diphenyltetrazolium bromide) technique and by mRNA cytokine expression of which IFN-γ and IL-4 are the most important against Capripoxvirus infection [70,71].

Since the three members of the genus *Capripoxvirus* present no serotypes and are genetically similar, it has been suggested the development of a universal vaccine to protect against the three viruses [116,153]. Thus far, there is no consensus on a universal vaccine—some strains can protect one species and induce lesions in another, and some are fully protective in homologous species and partially protective in other species (Table 2).

The lack of protection of SPV vaccines against goatpox has previously been reported by Prasad and Datt (1973) using SPV [159]. In Saudi Arabia, Abuelzein et al. (2003) [160] reported the appearance of the disease in goats vaccinated with SPV Romania vaccine locally produced; however, few data on vaccination coverage and vaccination conditions were presented. Although no challenge was performed, Abdelfatah et al. (2019) detected a satisfactory level of peripheral blood mononuclear cells (PBMCs) and lymphocytes in goats vaccinated with the Romania strain [158]. In a recent paper, Hamdi et al. (2020) established full protection of goats against the Romania strain in an experimental goatpox infection [57].

The use of SPV-based vaccines to protect cattle against LSV has been documented in Africa and the Middle East based on field observations. Studies carried out in Israel with RM65 strain demonstrated lower protection in cattle when vaccinated with the sheep dose [156] or at 10-times the sheep dose [21] when compared to the Neethling vaccine. In Jordan, a vaccination campaign was conducted in cattle using a vaccine based on the RM65 strain. The vaccine caused fever, reduced milk production, and the development of nodules in vaccinated cattle [161]. In Turkey, Sevik and Dorgan (2016) reported LSD vaccination failure with SPV Bakirkoy strain, at 3–4 times the sheep dose [115]. In Egypt, vaccination against LSDV in cattle was carried out with SPV Romania strain; however, cases of infection emerged in vaccinated herds as reported by Ali et al. (1990) [162], Abdallah et al. (2018) [163], and Zeedan et al. (2019) [164].

Investigations on the response of cattle vaccinated with Romania strain showed proliferation of lymphocytes and stimulation of gamma interferon and interleukin 4; however, no challenge has been conducted to test the efficacy of the vaccine [70,71,165]. Mikhael et al. (2016) observed, through serology and hypersensitivity testing, that the conferred protection by SPV Romania in cattle was insufficient [166]. In another study, Mikhael et al. (2017) tested a bivalent SPV Romania and GPV vaccine in cattle and assessed the humoral response by seroneutralisation, ELISA, and the cellular responses by stimulation of lymphocytes [167]. The authors concluded that the bivalent vaccine induced a better response than the monovalent Romania vaccine but recommended the use of a homologous strain. Similar results were reported by Aboul Soud et al. (2018) [168], who demonstrated that no serological response was induced in cattle vaccinated with the Romania strain, while a trivalent capripox vaccine (composed of SP Romania, GPV Held, and KSGP 0180) induced antibodies in 66% of vaccinated animals. In a recent experiment, partial protection was obtained in cattle vaccinated with Romania strain, no serology detected and at challenge, three out of seven vaccinated cattle showed viraemia and clinical signs similar to control animals [57].

The Gorgan goatpox strain was also tested in sheep, compared to the SPV vaccine at different doses, and challenged with a virulent SPV strain. Only sheep vaccinated with the SPV vaccine were protected and the authors, therefore, concluded that the Gorgan strain was unable to induce effective immunity in sheep against SPV infection [154].

In cattle, several studies showed the effectiveness of GPV strains to protect cattle against LSD. The Kedong and Isiolo strains, isolated in sheep in 1950 and identified as goatpox viruses [118], were able to protect vaccinated cattle against challenge [109]). The Gorgan strain, commonly used in Iran for vaccination against LSD, was tested in comparison with the KSGP O-180 strain and the Neethling strain in cattle. The Gorgan strain was more immunogenic than the two other strains at challenge [56]. However, the authors recommend undertaking large-scale studies to confirm their findings. Varshovi et al. (2017) [71] investigated humoral and cellular response in cattle after vaccination with Gorgan strain and found it immunogenic. In a recent experiment, complete protection in cattle was obtained by Zhugunissov et al. (2020) against challenge after the use of Gorgan strain at 10 times goat dose [169].

LSD vaccine based on Neethling has been used to protect sheep against SPV by Hamdi et al. (2020b); the authors report partial protection against virulent SPV challenge [57].

It appears that the consensus on one universal vaccine against the three capripoxvirus infections is not realistic. In addition, the geographic distribution of diseases is different. Thus, in some countries, the presence of SPV/GPV may or may not be accompanied by that of LSDV [2]. For instance, in North Africa, SPV is endemic, while no cases of LSD or GPV were reported. In southern Africa, LSD is endemic; however, SPV and GPV are absent. The European continent is free from GPV and SPV (except in Greece where outbreaks of SPV are still notified), while LSD has been reported in many countries of southeastern Europe. In the Middle East, central and eastern Asia, the three diseases are endemic. The use of a vaccine containing a virus not circulating in the country is not indicated [27].

Capripoxvirus infections are emerging diseases that represent a threat to ruminant industry farming in large geographical areas. Mixed flocks of sheep, goats, and cattle living in proximity is a common occurrence in endemic countries, and vaccination should be carried out systematically using the homologous vaccine, live or inactivated according to the epidemiological situation.

## Figures and Tables

**Table 1:** Capripoxvirus strains reported in the literature.

Isolate/Strain	Origin	Cells	Passages for Attenuation	Reference
SPV RM65	Sheep	Sheep kidney cells	30	[101]
SPV Romania	Sheep	Lamb kidney cells	40	[88]
SPV Bakirkoy	Sheep	Calf kidney cells	32	[102]
SPV Rumania Fanar	Sheep	Lamb testis	26	[103]
SPV Perego	Sheep	Lamb testis and calf kidney	11 times in lamb testis and 10 times in calf kidney	[104]
SPV Ranipet	Sheep	Sheep thyroid cells	35	[105]
GPV Gorgan	Goat	Kid kidney cortex cells	-	[106]
GPV Uttarkashi	Goat	Primary lamb testis cells and Vero cells	34 on primary cells and 26 on Vero	[107]
GPV Mysore	Goats	Primary lamb testis cells	25	[108]
GPV Kedong	Sheep	Lamb testis cells	-	[109]
GPV Isiolo	Sheep	Lamb testis cells	-	[109]
LSD KSGP O 180	Sheep	Bovine foetalmuscle cells	18	[110]
LSD KSGP O 240	Sheep	Lamb testis cells	13–27	[111]
LSD Neethling	Cattle	Lamb kidney cells and chorioallantoic membranes (CAM)	61 on primary cells and 20 on CAM	[99]

**Table 2:** Conferred protection by capripoxvirus vaccines.

Vaccine/Strain	Safety and Protection			Reference
	Sheep	Goat	Cattle	
GPV Gorgan	Safe, partially protective	Safe and protective	Safe and protective	[56,106,154]
GPV Mysore	-	Safe and protective	-	[108]
GPV Uttarkashi	-	Safe and protective	-	[107]
GPV Kedong and isiolo	-	-	Safe and protective	[155]
SPV RM65	Safe and protective	-	Partially protective	[21,156]
SPV Perego	Safe and protective	-	-	[103,157]
SPV Rumania Fanar	Safe and protective	-	-	
SPV Romania	Safe and protective	Safe and protective	Partially protective	[57,158]
SPV Bakirkoy	Safe and protective	-	Partially protective	[102,115]
LSD Neethling	Partially protective	-	Causes Neethling disease, protective	[23,57]
LSD KSGP 0180	Safe and protective	Safe and protective	Safe and protective	[110]
LSD KSGP 0240	Safe and protective	Safe and protective	Residual virulence, partial protection	[20,112,126,127]

-: not reported.
